# Excision of Anal Circumference with Hemorrhoids

**Published:** 1886-09-20

**Authors:** J. McF. Gaston

**Affiliations:** Professor of Surgery in the Southern Medical College, Atlanta, Ga.


### SOCIETY REPORTS.

### EXCISION OF ANAL CIRCUMFERENCE WITH HEMORRHOIDS.

BY J. McF. GASTON, M. D.,
Professor of Surgery in the Southern Medical College, Atlanta, Ga.

[READ BEFORE THE ATLANTA SOCIETY OF MEDICINE.]

A case of hemorrhoidal development in the form of an irregular vascular tumor protruding from the anus after evacuating the bowels, was referred to me on the 12th of August by Dr. C. A. Stiles, in the person of a gentleman who had suffered from the affection for several years. Upon examination, I observed that the several divisions of the mass were continuous throughout the circuit of the anus, and it was thought best to remove the entire protrusion by passing the chain of an ecraseur around the whole tumor, so as to excise the relaxed cutaneous tissue with the excrescence from the anus. There were no indurated fibrinous depositions in the protruded tissues, but an extreme turgescence of the structure under the constriction of the sphincter ani, indicating dilatation of the hemorrhoidal vessels.

A laxative of cream or tartar and sulphur having produced its appropriate effect, I proceeded to operate on the afternoon of the 13th, with the assistance of Drs. J. C. Olmsted, W. D. Bizzell, and W. L. Elkin. The A. C. E. mixture (alcohol 1 part, chloroform 2 parts, and sulphuric ether 3 parts), was used as the anæsthetic, after a subcutaneous use of morphine and atropine, with a satisfactory effect from the preliminary whisky toddy.

It was planned to pass up an ivory tube with a thick rubber tube over it within the anus, and constricting the tissues around this to effect a clear division with agglutination of the mucous and cutaneous tiusses by the action of the chain of the ecraseur; but, while tightening it, this tube slipped out, and the operation was completed without this guarantee for a complete separation of the tissues in a circular line. This resulted in a dragging upon the last portion after cutting nearly through, and retaining a thin layer of tissue in the grasp of the instrument, so that it was requisite to force it out, and, in so doing, the agglutinated margins of the adjacent structures became detached—which led to a free discharge of blood from the hemorrhoidal vessels. This ought not to have occurred, but for the necessary violence in relieving the imprisoned tissue, and I have performed this operation heretofore without any bleeding. Although the amount of hemorrhage did not seem to require the use of a styptic, it was thought prudent to resort to mechanical compression, and a plug of lint wrapped around the ivory tube already at hand was passed within the rectum, with the effect of arresting completely any appearance of blood externally. But some hours subsequently, upon visiting the patient, he manifested an inclination to have an evacuation, and, removing the tampon, coagula were expelled, showing that, while the constriction of the sphincter had prevented any escape around the obstructing plug, the blood had flowed internally. A plug was replaced, with the outer surface soaked with the tincture of persulphate of iron, which seemed for a time to arrest all hemorrhage, and I left the patient about 10 o'clock at night with an attendant, who was instructed to call me if the bleeding should recur. About midnight, a message being received that there was more hemorrhage, I found that in the meantime the tampon had escaped from the rectum, and profuse bleeding from the anal opening was kept up with marked prostration of the patient. Another plug was saturated with the styptic and bound firmly in the rectum by a perineal bandage secured to a belt around the waist. Having on a former occasion effectually stayed an alarming hemorrhage from the rectum after the division of an internal blind fistula by similar process, I felt great confidence in it, yet the powers of my patient being so nearly exhausted, I thought it might become necessary to resort to sanguineous transfusion or the injection of the saline solution into the veins, and sent for Dr. Elkin to come to my assistance with alcoholic stimulants, and such appliances as might be requisite. After remaining and watching the case together for more than an hour without any further return of the bleeding, he left me to a vigil of the greatest anxiety I have ever experienced over any patient during the many hairbreadth escapes witnessed in surgical practice.

A subcutaneous injection of morphine and atropine relieved restlessness, and a single dose of the fluid extract of ergot was given, with the free adminstration of whisky, and the addition of milk in the morning, to support the enfeebled powers of the patient.

My colleagues, Drs. Olmsted, Bizzell, and Elkin, were called in consultation, during the forenoon, as to the propriety of resorting to the intravenous saline injection, but as the patient seemed to be rallying, and there was no evidence of further bleeding, it was not considered necessary.

On the following afternoon there was a desire to evacuate the bowels, and upon releasing the fastenings of the plug, it was expelled with the discharge of a considerable amount of grumous blood, but no fresh bleeding ensued, and it was not thought requisite to replace the tampon.

There was the usual reactionary fever afterwards, and with increase of temperature reaching 104°, but with the use of quinine, malt, and milk punch for a few days, followed by claret, seltzer water, and compound decoction of Peruvian bark, all went on favorably in his general and local manifestations, so that three weeks after the operation he was in a condition to leave the city for his home, with a good prospect of a complete cure.

